# Processing of acoustic and phonological information of lexical tones in Mandarin Chinese revealed by mismatch negativity

**DOI:** 10.3389/fnhum.2014.00729

**Published:** 2014-09-16

**Authors:** Keke Yu, Ruiming Wang, Li Li, Ping Li

**Affiliations:** ^1^Center for Studies of Psychological Application, School of Psychology, South China Normal UniversityGuangzhou, China; ^2^College of International Culture, South China Normal UniversityGuangzhou, China; ^3^Department of Psychology and Center for Brain, Behavior, and Cognition, Pennsylvania State UniversityPennsylvania, PA, USA

**Keywords:** Chinese lexical tones, acoustic processing, phonological processing, mismatch negativity (MMN), preattentive stage

## Abstract

The accurate perception of lexical tones in tonal languages involves the processing of both acoustic information and phonological information carried by the tonal signal. In this study we evaluated the relative role of the two types of information in native Chinese speaker’s processing of tones at a preattentive stage with event-related potentials (ERPs), particularly the mismatch negativity (MNN). Specifically, we distinguished the acoustic from the phonological information by manipulating phonological category and acoustic interval of the stimulus materials. We found a significant main effect of phonological category for the peak latency of MMN, but a main effect of both phonological category and acoustic interval for the mean amplitude of MMN. The results indicated that the two types of information, acoustic and phonological, play different roles in the processing of Chinese lexical tones: acoustic information only impacts the extent of tonal processing, while phonological information affects both the extent and the time course of tonal processing. Implications of these findings are discussed in light of neurocognitive processes of phonological processing.

## Introduction

The use of lexical tones to differentiate lexical semantics is a characteristic of Chinese and other tonal languages. According to Yip (2002), the world’s languages can be categorized into three types depending on the role that the pitch information plays in the expression of meaning: tone language (e.g., Chinese), intonation language (e.g., English), and pitch-accent language (e.g., Japanese). In languages like Chinese, different lexical semantics are expressed through the variations of pitch height and pitch contour at the syllable level, as opposed to intonation languages where pitch variations occur only at the phrase or sentence level, or pitch-accent language where pitch variations occur between syllables. In recent years, researchers have become interested in the neurocognitive processes associated with tonal languages (see Gandour, 2006; Jongman et al., 2006 for reviews). Several studies have also used neuroimaging techniques including event-related potential (ERP) and functional magnetic resonance imaging (fMRI) to study the processing of lexical tones in Chinese (e.g., Gandour et al., 2000; Klein et al., 2001; Li et al., 2008; Zhang et al., 2011; Wang et al., 2013a).

When a native speaker processes lexical tones in Chinese,^1^ the speaker deals with at least two types of information: the acoustic information that includes the physical features of auditory input such as the fundamental frequency (F0), and the phonological information that expresses lexical semantics on the basis of which word categories are identified (Luo et al., 2006; Xi et al., 2010). To investigate the difference between the processes of the acoustic vs. the phonological information in lexical tones, one line of previous research has been to understand whether lexical tonal processing in Chinese is left or right lateralized in the brain. The functional hypothesis claims that when tones are processed as phonological units they are lateralized to the left hemisphere, whereas when they are processed as purely acoustic information they are lateralized to the right hemisphere (van Lancker, 1980; Wong, 2002). Chinese lexical tones contain both the acoustic information and the phonological information. Thus, based on this hypothesis, Chinese tonal processing involves both hemispheres for different processing of these two kinds of information. A competing theory, the acoustic hypothesis, claims that the brain lateralization of tonal processing depends on the acoustic properties of the auditory input. Spectral variations such as those contained in pitch information are preferentially processed in the right hemisphere, whereas temporal variations such as those contained in vowels are processed more strongly by the left hemisphere (Zatorre and Belin, 2001; Zatorre et al., 2002). According to this hypothesis, the acoustic contrasts and the phonological contrasts in Chinese tones are both spectral variations, and so both acoustic processing and the phonological processing are lateralized to the right hemisphere.

In light of these views on brain lateralization, Gandour et al. have put forward a comprehensive hypothesis on this issue (Gandour et al., 2000, 2004; Gandour, 2006). They suggested that lexical tonal processing engages both hemispheres, depending on the types of information involved during processing. In particular, for the same lexical tones, the left hemisphere is more involved in the semantic processing whereas the right hemisphere more in the acoustic processing of the pitch information. Given that processing of lexical tones involves the processing of both acoustic information and phonological information (which is semantically differentiating), this hypothesis suggests that there is no simple lateralization pattern associated with the brain’s processing of tones.

The key issue under consideration here is therefore whether processing involves acoustic features of tones or phonological features of tones. Luo et al. (2006) used mismatch negativity (MMN) to study the features of tonal processing. MMN is a powerful method to examine the early stages of acoustic and phonological processing, as previous studies have indicated (e.g., Näätänen et al., 1978, 2007; Näätänen and Alho, 1997). It is an eletrophysiological component that reflects the brain’s automatic detection of deviant patterns that do not match the general pattern that has been observed, which peaks at typically around 200–250 ms after stimulus onset, mostly in the frontocentral areas. MMN responses can be elicited by an oddball paradigm in which several infrequent deviant stimuli are embedded in frequent standard stimuli during auditory presentation. Luo et al. (2006) found that the mean amplitude of MMN elicited by tone deviants in the right hemisphere was larger than that in the left hemisphere. Their conclusion was that at the pre-attentive stage, a stage that is characterized by automatic processing when people are unconscious about the detailed properties of the stimuli (Kubovy et al., 1999), listeners mainly process the acoustic information of lexical tones. At this stage, the processing is usually lateralized to the right hemisphere. At a second stage of tone processing, the attentive stage, a stage in which people process the presented stimuli consciously, listeners tended to process the semantic information of tones via the left hemisphere. Luo et al.’s novel finding was that, in addition to the work of Gandour et al. whether the cognitive processing of Chinese lexical tones involves both hemispheres will depend on the different processing stages. Thus, the MMN provides an excellent measurement of the time course of processing, contributing to additional insights in lexical tone processing.

In addition to the lateralization debate of tone processing in Chinese, recent studies have also highlighted the role of categorical perception of tones. Categorical perception refers to the ability that human listeners can perceive continuous acoustic signals as discrete linguistic representations: listeners are sensitive to the boundaries between different phonetic categories, but are insensitive to acoustic changes within the boundaries of same phonetic category (Liberman et al., 1957, 1967). This across-category vs. within-category perception difference has been extensively studied in previous work with segmental phonemes, such as consonants and vowels (e.g., with VOT characteristics), but are less well understood in studies with suprasegmental features such as tones. It has been found recently, however, that the perception of lexical tones shows categorical perception just as do phonemes: native speakers of tonal languages are more sensitive to across-category tonal variations than within-category variations (see Francis et al., 2003; Hallé et al., 2004; Xu et al., 2006; Xi et al., 2010).

Xi et al. (2010) used MMN to examine categorical perception of tones in Chinese. The authors found that although both across-category and within-category variations of tones elicited MMNs in bilateral frontal-central areas, the former elicited larger MMNs in the left than that in the right hemisphere, whereas the latter elicited larger MMNs in the right than in the left hemisphere. These patterns provide support for the categorical perception of tones, while at the same time evidence for the hypothesis that both hemispheres are involved in the processing of tones (Gandour, 2006), in that the processing of within-category stimuli mainly involves the acoustic processing of pitch information while the processing of across-category stimuli the phonological information. Moreover, within the time window of MMN (200–250 ms), both acoustic information and phonological information are processed in parallel, which contrasts to the two-stage hypothesis of Luo et al. (2006).

An fMRI study by Zhang et al. (2011) further indicated that the interaction between acoustic processing and phonological processing in the two hemispheres: across-category variations elicited stronger activation in the left middle temporal gyrus than did the within-category variations, whereas within-category variations elicited stronger activation in the right superior temporal gyrus. These fMRI findings indicate how low-level acoustic analysis (within-category) is modulated by high-level phonological representations (across-category), and are therefore consistent and complementary with the MMN findings reported in Xi et al. (2010). In another ERP study of categorical perception of tones at the attentive stage, Zhang et al. (2012a) found that the conscious processing of the within-category stimuli and the across-category stimuli involved the N2b and P3b components. The data were compatible with the response patterns as Xi et al. (2010): for both N2b and P3b, across-category stimuli elicited larger response in the left recording sites than the right; while the within-category stimuli elicited the same response in both hemispheres.

Such findings of categorical perception of tones also have significant implications for understanding disorders during processing. For example, Zhang et al. (2012b) found that Chinese-speaking children who were at risk for dyslexia showed no significant differences between the across-category vs. within-category stimuli, in contrast to both adults and age-matched normally developing children. It could be that children with reading disorders may perceive the phonological information in the same way as they do with acoustic information.

One important question that remains unclear from previous research is whether acoustic information and phonological information of tones are fundamentally different (i.e., different kinds of information), or whether they are only different on a continuum (same kinds of information). As suggested by the functional hypothesis (van Lancker, 1980), the pitch information contained in Chinese lexical tones can be divided into two types depending on whether it serves as acoustic signal or phonological unit: these are two distinct kinds and also have different cognitive processing consequences. Gandour et al. (2000) further distinguished between the pure acoustic features vs. acoustic features plus semantic features in the perception of tones such as lexical tones in Chinese and Thai. An alternative view to the above is that there is no fundamental difference between the two in terms of cognitive processing: the pitch contrasts in tones are spectral variations, and the processing of these contrasts always involves the same type of acoustic analysis regardless of its specific features (e.g., Zatorre and Belin, 2001; Warrier and Zatorre, 2004; Ren et al., 2009). This alternative view suggests that the fundamental features of the acoustic information and the phonological information both lie in the spectral variations, and therefore are similar with regard to neural mechanisms, the processing of phonological information and that of acoustic information involve the same neural patterns, both in the right hemisphere. This argument contrasts with the suggestion of different neural correlates of acoustic vs. phonological processing, as discussed above (e.g., MMN and fMRI evidence of left vs. right lateralized patterns).

In the present study, we designed an experiment to further explore the different roles that acoustic vs. phonological information may play in the perception of Chinese tones. In particular, we relied on the MMN patterns that have been previously used successfully in this area (Luo et al., 2006; Xi et al., 2010). Previous work, including Xi et al. (2010) and Zhang et al. (2011), has examined only the contrast between acoustic information and phonological information as the contrast between within-category and across-category variations. In the MMN paradigm, this means that the across- or within-category deviants are equally spaced when compared with the standard stimuli of tonal contrasts. In the current study, to further differentiate the two types of information, we added a new variable, the acoustic interval, which refers to the different F0 interval between the deviant and the standard stimuli. This new variable has two levels, small acoustic interval vs. large acoustic interval.

**Figure 1:**
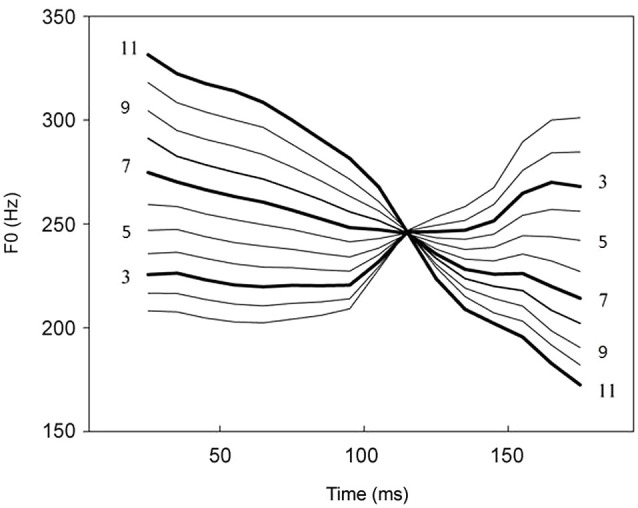
**Tone contours of the continuum from /ba2/ to /ba4/ (from Xi et al., 2010; reproduced with permission from authors and publisher)**. Stimuli 3, 5, 7, 9 and 11 are marked. Continua 3, 7 and 11 marked with thick lines were used in previous study (Xi et al., 2010; Zhang et al., 2011, 2012a,b). In the present study, continua 5, 9 were also chosen to test the acoustic interval variable.

The stimuli materials used by Xi et al. (2010) and Zhang et al. (2011) were chosen from a Chinese lexical tonal continuum, from the high-rising tone (tone 2,/pa2/) to the failing tone (tone 4,/pa4/). The differences between the two tones can be acoustically manipulated according to the F0 contour, to produce a range of in-between tones, resulting in a continuum of stimulus 1 to stimulus 11 (see Figure 1 and Section Materials and Methods for details). Previous studies were focused on categorical perception of tones, so only stimuli 3, 7, 11 were used in their experiments (e.g., Xi et al., 2010; Zhang et al., 2011). In the current study, we continued to use the 11 stimulus set, but we included stimuli 5 and 9 in our experiment, to produce an orthogonal design that involved both phonological categories and acoustic intervals (see Section Materials under Methods for details). This design would allow us to systematically test the role of acoustic vs. phonological information in the processing of Chinese lexical tones, with both small and large interval differences in the acoustic signal and across and within differences in the phonological category. Specifically, we predict that the MMN patterns may reflect different impact of these two types of information, with regard to both the magnitude and the peak latency. We hypothesize that if the acoustic and the phonological information belongs to the same type of information (different dimensions of the auditory input), as proposed by Zatorre et al. the variations in the MMN mean amplitudes and peak latencies would be similar. On the other hand, if these two types of information are fundamentally different and reflect different cognitive processes, we may see variations of MMN patterns as a function of their differences, for example, in amplitude and time course.

## Materials and methods

### Participants

Thirty-six neurologically healthy volunteers (21 females, mean age 20 years, range 19–21 years) took part in the study. All participants had normal hearing and minimal musical experience, and were native speakers of Mandarin Chinese recruited from the South China Normal University. The participants were all right-handed basing on their self-assessments. They gave written consent before they took part in the experiment and received monetary compensation for taking part in the experiment. This study was approved by the ethics review board of South China Normal University.

### Experimental design

Our study used a within-subject design with two factors: phonological category (within-category/ across-category), and acoustic interval (large/small). See *Materials* section below for details. The dependent variables were the mean amplitude and the peak latency of the MMNs elicited by the stimuli.

### Materials

The materials were the same as used in Xi et al. (2010; see Figure 1), except noted below. They were acoustically manipulated based on Chinese lexical tones, to produce a continuum from the high-rising tone (tone 2) to the failing tone (tone 4). Two Chinese monosyllables, /pa2/and /pa4/, were firstly produced by a native speaker and digitally edited by Sound-Forge (SoundForge9, Sony Corporation, Japan) to get a same duration (200 ms). Then the two monosyllables were further edited by the Praat software^2^ to keep the same acoustic features except the pitch contour. They were then used as the endpoint stimuli to create the 10-interval lexical tone continuum in Matlab (Mathworks Corporation, USA) using the toolbox of STRAIGHT (Kawahara et al., 1999). Thus there were 11 artificially generated stimuli in the continuum that differed only in the F0 (labeled as stimulus 1 to stimulus 11). The F0 intervals between any two adjacent stimuli were also acoustically manipulated to be the same, for the purpose of the experiment.

In Xi et al. (2010) study, stimuli 3, 7, and 11 were used to construct the across-category pair (3 vs. 7) and the within-category pair (7 vs. 11) in their experiment. These pairs of contrast between 3 and 7, and 7 and 11, had large acoustic intervals for both across- and within-category contrasts. Given the focus of the previous studies on categorical perception, they served to address the research questions well. In the present study, we constructed an orthogonal design incorporating two variables, phonological category (as in Xi et al., 2010), and acoustic interval, in the following way: stimuli 5 vs. 7 (small interval, across category), stimuli 3 vs. 7 (large interval, across category), 7 vs. 9 (small interval, within category), stimuli 7 vs. 11 (large interval, within category). This new design would allow us to address additional research questions, as discussed in the Introduction section.

To ensure that the materials meet the need of our ERPs experiment, we conducted a norming study for the experimental material with a separate group of participants. 15 participants took part in an identification task and a discrimination task. In the identification task, they were asked to identify whether each stimulus (stimulus 3, 5, 7, 9, 11) was the high-rising tone (tone 2) or the failing tone (tone 4). 20 trials for each stimulus were randomly presented in isolation, with no stimulus being presented consecutively three times. In the discrimination task, participants were asked to judge whether a pair of presented stimuli were the same or not. The stimulus pairs were presented randomly, and the pairs which contained different stimuli were presented in both directions, each for 10 trials. To balance the number of “yes” or “no” responses, we included pairs of the same stimulus, each presented for 16 times (80 in total). Practice trials were given to the participants before the actual norming experiment.

### Procedure

An improved passive oddball paradigm, proposed by Duncan et al. (2009), was used for this experiment. Classic oddball paradigms usually contained one kind of deviant stimuli and one kind of standard stimuli in a block, whereas the improved paradigm contained more than one kind of deviant stimuli in a block. The improved oddball paradigm has been shown to produce MMNs more quickly and effectively. A total of 1015 stimuli were presented to the participants during the experiment, including 15 standard stimuli at the beginning of the experiment and followed by 600 standard stimuli and 400 deviant stimuli, each type of deviant being 100. 15 standard stimuli were firstly presented to promote the participants to adapt to the experiment. The deviants were presented pseudo-randomly among standards, and any two adjacent deviants were different. Each stimulus was presented for 200 ms. The stimulus-onset-asynchrony (SOA) was set to be 800 ms. The stimulus presentation lasted for about 15 min.

Participants took a passive auditory task. They were instructed to watch a movie (Whisper of the Heart) (e.g., Duncan et al., 2009) in which the sound tracks of the movie were turned off. Moreover, they saw the movie for 5 min first before starting the experiment. No overt responses were required of the participants. To ensure that the participants paid attention to the movie, the experimenter asked them to answer five questions about the movie after the experiment. The experiment lasted 22 min in total (5 min in movie plus 15 min in experimental session proper).

### Electroencephalogram (EEG) recording

EEG was recorded using a 64-channel (Ag–AgCl) NeuroScan system (NeuroScan). Electrodes were positioned following the 10–20 system convention. The reference electrode was placed at the tip of the nose and the ground electrode was placed at FPz. The vertical electro-oculogram (EOG) was recorded supra- and infra-orbitally from the left eye. The horizontal EOG was recorded as the left vs. right orbital rim. The impedance of each electrode was kept below 5 kΩ. EEG and EOG signals were digitized online at 500 Hz and band-pass filtered from 0.05 to 100 Hz.

### Data analysis

Off-line signal processing was carried out using Scan 4.3 (NeuroScan). The reference electrode was converted first to bilateral mastoid (M1 and M2) and some artifacts were rejected manually. Data from two participants were excluded from further analyses due to their excessive eye blinking. Data were then adjusted by eliminating the interference of the horizontal and vertical eye-movements. The data were segmented for a 700 ms time window, including a 100-ms pre-stimulus baseline. Then the baseline was corrected according to Zhao (2010) and the recorded trials with eye blinks or other activities beyond the range of −80 to 80 mV were rejected. The data from the whole-head recordings were off-line band-pass filtered (1–30 Hz) with a finite impulse response filter. Finally, the ERPs evoked by the standard stimuli and the deviant stimuli were calculated by taking the averages of individual trials from each subject. Only those data with at least 80 accepted deviant trials in each deviant condition were adopted. With this criterion, data from another two participants were excluded from further analyses. MMNs were then derived by subtracting the ERPs evoked by the standard stimuli from those evoked by the deviant stimuli.

On the basis of findings from previous studies in the literature, we selected three recording sites for statistical analyses: F3, F4 and FZ. In previous studies of MMN, the MMN component typically peaks around 200–250 ms (Näätänen et al., 1978, 2007; Näätänen and Alho, 1997). Researchers usually choose a time window around this peak based on the grand-average waveforms of their particular data, which could include 100–350 ms (e.g., Kaan et al., 2008), 150–300 ms (e.g., Tsang et al., 2011), 230–360 ms (Wang et al., 2013b), and so on. Considering the usual range of MMN’s peak and the grand-average waveforms of the present study, we chose a time window of the MMNs to be 200–350 ms. The peak of MMN for each subject at the four conditions in this time window was detected by using the procedure “Peak detection” in Scan 4.3 (NeuroScan). The MMN mean amplitudes were calculated by averaging the responses within the time window ranging from 20 ms before the peak of MMN recorded from electrode FZ to 20 ms after that peak. The mean amplitudes and peak latencies of the three chosen recording sites (F3, F4, FZ) were used for further statistical analyses.

## Results

### Norming experiment

In the identification task, participants were asked to identify whether each stimulus (stimulus 3, 5, 7, 9, 11) was the high-rising tone (tone 2) or the failing tone (tone 4). In the discrimination task, participants were asked to judge whether a pair of presented stimuli were the same or not. We analyzed the proportions of different tone judgments in the identification task and the proportions of “yes” or “no” responses in the discrimination task according to Xi et al. (2010) study. In the identification task, the proportion of different stimuli, 3, 5, 7, 9, 11, which were regarded as tone 4, was 4.4%, 8%, 87.8%, 97.4%, 98.2%, respectively. These results showed that the participants identified stimuli 3 and 5 as tone 2 and stimuli 7,9,11 as tone 4 indeed. In the discrimination task, the proportion of negative judgments for pairs 3–7, 5–7, 9–7, 11–7 was 89.7%, 89.7%, 15%, 30%, respectively. These results showed that the participants identified stimuli 3 and 7, and 5 and 7 as different tones, and stimuli 7 and 9, and 7 and 11 as the same tone.

The norming experiment showed that we can reliably treat stimuli 3 and 7, 5 and 7 as across-category pairs and stimuli 7 and 9, and 7 and 11 as within-category pairs. Considering the F0 intervals as illustrated above, stimuli 3 and 7, 11 and 7 were treated as the large interval stimulus pairs, and the F0 intervals of each pair were four F0 units. Similarly, stimuli 5 and 7, 9 and 7 were treated as the small interval stimulus pairs, and the F0 intervals of each pair were two F0 units. These results helped us to determine stimulus 7 in the tonal continuum (Figure 1) as the standard stimulus, stimulus 3 as an across-category with large interval deviant, stimulus 5 as an across-category with small interval deviant, stimulus 9 as a within-category with small interval deviant, and stimulus 11 as a within-category with large interval deviant.

### ERP experiment

**Figure 2:**
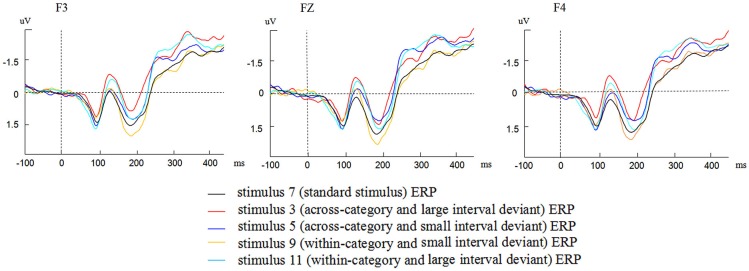
**Grand average waveforms elicited by the standard stimulus and four deviants at F3, FZ, F4 electrode locations**.

Figure 2 presents the grand average waveforms of the ERPs elicited by the standard stimulus and four deviants at F3, FZ, F4 electrode locations. As shown in Figure 2, different waveforms of the ERPs to the standard stimuli and the four deviants were observed at the three electrode locations.

To ensure that the deviant stimuli elicited MMNs, we conducted four paired samples *t*-tests to compare the grand average amplitudes of the four deviant contrasts in the time window of MMN at three electrode locations. The difference between the grand average amplitudes of stimuli 3,5,11 and standard stimuli were significant (*t*_(1,31)_ = 6.124, *p* < 0.001; *t*_(1,31)_ = 3.928, *p* < 0.001; *t*_(1,31)_ = 2.625, *p* = 0.013 < 0.05). The difference between stimuli 9 and standard stimuli was marginally significant (*t*_(1,31)_ = 1.718, *p* = 0.096 < 0.1). Thus the four deviant stimuli elicited reliable MMNs.

**Figure 3:**
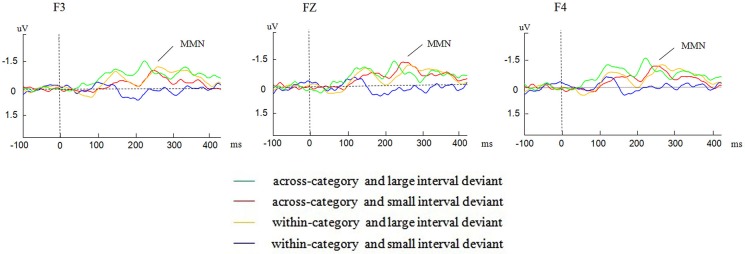
**Grand average traces of MMN evoked by different deviants from the F3, FZ, and F4 electrode locations**.

Figure 3 presents the different MMNs obtained by subtracting the ERP waveforms of the standard stimuli from those of the deviant stimuli at F3, FZ, F4 electrode locations. In the figure, distinct MMNs were displayed in the MMN time window (200–350 ms) at the three electrode locations.

We conducted two separate repeated-measures 2 × 2 ANOVAs, with phonological category (within-category/across-category) and acoustic interval (large/small) as independent variables, one for the mean amplitude and another for the peak latency of the MMNs. For all analyses, degrees of freedom were adjusted according to the Greenhouse–Geisser method when appropriate.

#### MMN peak latency

**Figure 4:**
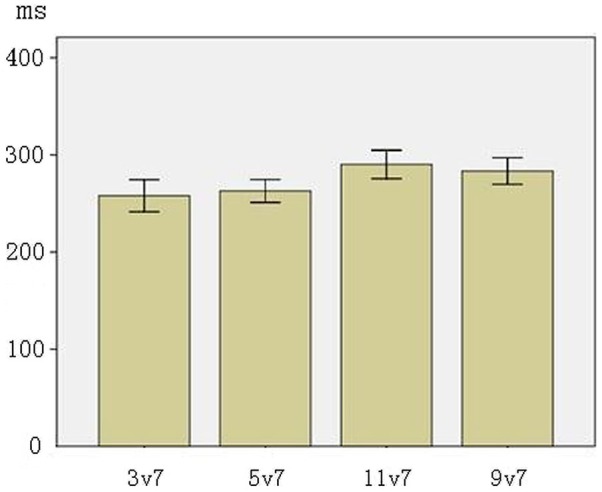
**The average of MMN peak latencies in F3, F4, FZ electrodes (The error bars represent standard errors)**.

Figure 4 presents the mean peak latencies of MMNs at F3, F4, FZ electrodes (the line segment represents one standard error). ANOVA results showed a significant main effect of phonological category (*F*_(1,31)_ = 15.256, *p* < 0.001, across-category < within-category), reflecting that the across-category deviants were processed earlier than the within-category deviants. There was no significant main effect of acoustic interval, nor an interaction between phonological category and acoustic interval (*p*s > 0.10).

#### MMN mean amplitudes

**Figure 5:**
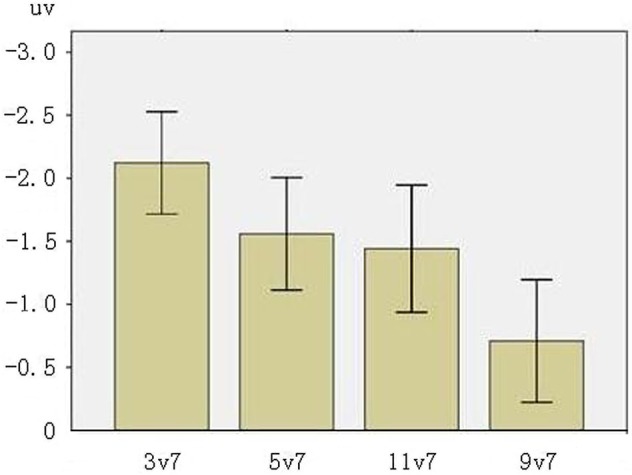
**MMN mean amplitudes in F3, F4, FZ electrodes (The error bars represent standard errors)**.

Figure 5 presents the mean peak amplitudes of MMNs at F3, F4, FZ electrodes. The main effect of phonological category was significant (*F*_(1,31)_ = 20.312, *p* < 0.001, across-category > within-category), showing that the processing of across-category deviants elicited greater MMN patterns than the processing of within-category deviants. There was also a significant main effect of acoustic interval (*F*_(1,31)_ = 9.924, *p* < 0.01, large interval > small interval), indicating that the processing of large interval deviants elicited greater MMN patterns than the processing of small interval deviants. There was no significant interaction between phonological category and acoustic interval (*p* > 0.10).

## Discussion

The perception of lexical tones in Chinese consists of the processing of the acoustic and the phonological information. But it has been debatable in the literature whether these two types of information are fundamentally different or whether they belong to the same auditory input that involves similar cognitive processes. As we reviewed earlier in the Introduction section, previous studies have investigated the differences between the acoustic information and the phonological information from the perspective of brain lateralization (Xi et al., 2010; Zhang et al., 2011, 2012a). The present study has focused on the different roles of acoustic vs. phonological information in native Chinese listener’s processing through acoustic interval and phonological category. In particular, we used a paradigm from the MMN studies of categorical perception of Chinese lexical tones, examining four kinds of stimuli with different phonological categories (across vs. within) and distinct acoustic intervals (large vs. small).

The results from this study show that the acoustic and the phonological information of Chinese lexical tones have distinct impacts on processing as revealed by MMNs. The main effect of phonological category and acoustic interval for MMN mean amplitudes are both statistically significant. In particular, MMNs elicited by across-category deviants were larger than those by within-category deviants and MMNs in response to large interval deviants were greater than small interval deviants. The results revealed that both types of information influenced MMN mean amplitude. But only phonological category showed a significant main effect for MMN peak latency: the MMN peak latencies for the across-category deviants were shorter than for the within-category deviants.

In the ERP literature, the mean amplitude of ERP components has been used to reflect the extent of neural resources during cognitive processing, and the peak latency has been argued to indicate the time course of the process (e.g., Duncan et al., 2009). The present study found that the acoustic information with different intervals only affected the MMN mean amplitude, but the phonological information influenced both the mean amplitude and the peak latency of the MMNs. The results revealed that the acoustic information of Chinese lexical tones impacted the extent of tonal processing while the phonological information affected not only the extent but also the time course of tonal processing. These patterns showed the different roles that the acoustic information and the phonological information play in the processing of Chinese lexical tones at the pre-attentive stage.

As discussed earlier, Gandour et al. have argued for the functional hypothesis of tonal processing, according to which the phonological information contained in pitch information, such as in tones, is different from the general acoustic information, because the former expresses lexical semantic differences (e.g., Gandour et al., 2000, 2002; Wong, 2002). An alternative view, the acoustic hypothesis proposed by Zatorre et al. perceives the pitch information in tones as no different from general acoustic information (e.g., Zatorre and Belin, 2001; Warrier and Zatorre, 2004). In the present study, distinct profiles in MMN mean amplitudes and peak latencies were observed to be associated with the acoustic and the phonological variations. These results provide support to the functional hypothesis rather than the acoustic hypothesis.

Some previous studies such as Luo et al. (2006), proposed that acoustic information of tones is mainly processed at the pre-attentive stage while phonological information is processed at the attentive stage. The proposal agreed with the view that the processing of the acoustic information and the phonological information are different. However, this proposal emphasized that the two kinds of information are processed at different stages. It is generally agreed that people can automatically process the stimuli without attention at the pre-attentive stage. Recent studies, in contrast to Luo et al. (2006), have suggested that acoustic and phonological information may be processed at both attentive and pre-attentive stages in parallel (Xi et al., 2010; Zhang et al., 2012b). In the present study, we differentiated the acoustic and the phonological information with a more fine-grained level, especially with regard to the acoustic interval of the stimulus. That is, the stimuli contained not only phonological category differences (different tones), but also acoustic interval changes (same tone with different F0). The results showed that both the acoustic and the phonological information of Chinese lexical tones can be processed at pre-attentive stage by native listeners. In addition, the present study found that the peak latencies of MMNs elicited by the across-category deviants were earlier than by the within-category deviants. Thus, phonological information associated with lexical tones may be processed even before the acoustic information at pre-attentive stage. This is a rather surprising finding that directly contrasts with arguments of the two-stage model by Luo et al. (2006).

How do we account for the surprising finding that phonological information may be activated even earlier than acoustic information in Chinese lexical tone processing? As discussed above, MMN reflects the automatic detection of distinct stimuli, acoustic or phonological, by the human brain. Considering the auditory stimuli in the present study, the within-category stimuli differed only in the acoustic information, while the across-category stimuli involved differences in both the phonological and the acoustic dimension. Thus, MMN may reflect the latter differences more easily and quickly, which is why we observed shorter peak latency of the across-category stimuli.

Some researches considered that the MMNs elicited by tone variations may result from the long-term memory trace of Chinese lexical tones. Chandrasekaran et al. (2007a) assessed the different MMN responses to non-speech stimuli involving similar pitch variations as Chinese lexical tones by Chinese and English listeners. Their results showed that Chinese listeners had larger MMN responses than English listeners, which indicated that the same non-speech stimuli involving pitch variations may activate long-term memory traces of lexical tones for Mandarin listeners (Chandrasekaran et al., 2007a). The patterns from the present study showed that the MMNs elicited by the stimuli involving both semantic variations and acoustic variations were larger and earlier than the stimuli only involving acoustic variations in mean amplitude. It seems that long-term memory representation of tones may have contributed to these patterns. Thus, based on Chandrasekaran et al.’s finding and our own results, the activation of the long-term phonological memory trace may play a significant role to enhance tone perception with regard to the processing of phonological information.

Pitch contour refers to the direction of change in F0 according to Gandour (1983). Chandrasekaran et al. (2007b) investigated whether different pitch contours of Chinese lexical tones have different impacts on tonal processing by tone 1, tone 2 and tone 3. Their results showed that the MMN peak latency elicited by tone 1 vs. tone 3 was earlier than that elicited by tone 2 vs. tone 3. As the difference between tone 1 and tone 3 was larger than that between tone 2 and tone 3 in pitch contour shapes, they concluded that the different pitch contour shapes impacted the MMN peak latencies. In the present study, we compared the within-category stimuli (9 vs. 7; 11 vs. 7) with the across-category stimuli (3 vs. 7; 5 vs. 7), which have different direction of pitch contour (see Figure 1). That is, the difference between the across-category stimuli and the standard stimuli was larger than that between the within-category stimuli and the standard stimuli in pitch contour shapes. Our result showed that the peak latency of across-category deviants was earlier than that of within-category deviants. This pattern is consistent with Chandrasekaran et al. (2007b) and further indicated the impact that pitch contour shapes have on the MMN peak latency. In addition, as discussed earlier, the present study further indicated the interaction of two independent variables, showing the different impacts of acoustic interval and phonological category in lexical tone processing. However, there remains the question of whether the changes in pitch contour shapes and the changes in phonological information have the same amount of impact on tonal processing. Our current study design cannot yet address this question and it should be examined in future research.

In conclusion, our study has explored MMN patterns (mean amplitude and peak latency) to identify the cognitive processes associated with the pre-attentive stage processing of both acoustic and phonological information involved in the perception of Chinese lexical tones. The results showed that the acoustic information of tones only impacts the extent of tonal processing, whereas the phonological information impacts the time course as well as the extent of the processing. Our data suggest that the acoustic information and the phonological information of tones were distinct auditory inputs, at least to the native listeners of Chinese who have long-term experience with the representation of pitch information that differentiates lexical meanings.

## Conflict of interest statement

The authors declare that the research was conducted in the absence of any commercial or financial relationships that could be construed as a potential conflict of interest.

## Footnotes

^1^Although our experimental materials involve lexical tones only from Mandarin Chinese, the findings from this study apply to all Chinese dialects. We therefore use “Chinese” as a generic term henceforth in the paper to refer to all Chinese dialects.

^2^http://www.fon.hum.uva.nl/praat/
